# Paraldehyde in the Treatment of Asthma

**Published:** 1899-02-18

**Authors:** 


### PARALDEHYDE IN THE TREATMENT OF ASTHMA.

Dr. Alexander MacGregor draws attention to paraldehyde as a drug, which in his hands has done good service in allaying the paroxysms in spasmodic asthma and in procuring good sleep. He says: "Paraldehyde is absolutely safe. It not only relieves the spasm, but it induces tranquil, refreshing sleep, without any objectionable after effects. Besides, no evil results follow a prolonged use of paraldehyde; it does not give rise to a habit, and on this account is a much more desirable drug than morphine or chloral." He gives details of a series of cases in which he has employed this drug, and it certainly would appear that he has fully proved its utility. Dr. MacGregor points out that although in many cases iodide of potassium and tincture of lobelia do much to relieve bronchitis and to lessen spasm, their effect is immensely increased by securing sleep and preventing nocturnal spasms by means of paraldehyde. The drug occasionally causes sickness, and its disagreeable pungent taste makes it objectionable to children and nervous patients, but it is well disguised in cinnamon water and tincture of orange peel. As a rule it acts so rapidly that it is well that the dose should be taken after going to bed. For adults it is best to begin with a dose of 60 minims.

